# Effect of different shielding conditions on the stability of Cisplatin

**DOI:** 10.1186/s40780-020-00163-x

**Published:** 2020-03-11

**Authors:** Tomoya Abe, Daigo Matsumoto, Toshiaki Nakayama, Yukinari Shimazaki, Atsunobu Sagara, Dan Kanehira, Takuya Azechi, Fumiaki Sato, Hiroyasu Sakai, Tetsuro Yumoto, Junzo Kamei

**Affiliations:** 1 Department of Pharmacy, Saitama Children’s Medical Center, 1-2 Shintoshin, Chuo-ku, Saitama, Japan; 2 Division of Pharmacy Professional Development and Research, Hoshi University School of Pharmacy and Pharmaceutical Sciences, 2-4-41 Ebara, Shinagawa-ku, Tokyo, Japan; 3 Department of Pharmacy, NTT Medical Center Tokyo, 5-9-22 Higashi-Gotanda, Shinagawa-ku, Tokyo, Japan; 4 Division of Applied Pharmaceutical Education and Research, Hoshi University School of Pharmacy and Pharmaceutical Sciences, 2-4-41 Ebara, Shinagawa-ku, Tokyo, Japan; 5 Department of Biomolecular Pharmacology, Hoshi University School of Pharmacy and Pharmaceutical Sciences, 2-4-41 Ebara, Shinagawa-ku, Tokyo, Japan

**Keywords:** Cisplatin, Stability, Shading cover, pH, HPLC, Chemotherapy

## Abstract

**Background:**

Because cisplatin (CDDP) decreases upon light exposure, it is necessary to prevent such exposure during administration. However, the shielding conditions employed are not uniform. Therefore, in this study, we examined the shielding effects of four shading covers, which are commonly used to ensure the stability of CDDP in clinical settings.

**Methods:**

Four shielding conditions, along with a control, were tested under a 1000-Lux white fluorescent lamp at room temperature: aluminum foil (Al), brown shading cover (BSC), yellow shading cover (YSC), milky-white anti-exposure cover (MAC), and no shading cover (NSC). Under each shielding condition, the relationship between the wavelength and transmittance was monitored in the range of 200–800 nm. CDDP was diluted to three concentration levels: 50, 100, and 250 μg/mL. Furthermore, the amount of remaining CDDP and the pH in the solutions were measured for 120 h.

**Results:**

We found that BSC, YSC, and MAC conditions allowed various levels of transmittance; however, Al could not completely transmit light at all wavelengths. Moreover, we showed that the CDDP decreased under MAC and NSC conditions in a time-dependent manner, whereas this decrease was prevented under Al, BSC, and YSC conditions till 120 h. We also demonstrated increases in pH under MAC and NSC conditions in a time-dependent manner, which was prevented under Al, BSC, and YSC conditions till 120 h. Similar results were observed for all three CDDP concentration levels. The results also indicated the approximate relationship between the amount of remaining CDDP and the pH increase.

**Conclusions:**

Considering the opacity of each cover, our results suggest that BSC and YSC are useful and effective for minimizing CDDP degradation in clinical settings. Our results also indicate the alternatives for preparing, storing, and administering CDDP in clinical facilities, making the treatment schedule more flexible. Cumulatively, these findings indicate that the use of the appropriate shading covers, such as BSC or YSC, prevents the decrease in CDDP under fluorescent lighting, potentially contributing to achieving its full therapeutic effect.

## Background

Cisplatin (CDDP), a platinum-based drug, is the most potent anti-cancer agent and is widely used for cancer treatment [1]. In particular, it plays crucial roles in treating lung, gastric, and ovarian cancers [2–4]. The dosage and administration of CDDP have been determined in accordance with certain regimens for each type of cancer. For anti-cancer agents such as dacarbazine, it has been reported that the provision of appropriate shielding conditions affects the treatment and patient’s quality of life [5]. Such shielding is important to maintain the stability of the drugs because of the differences in the drug preparation method and administration schedule depending on the regimen. Regarding the stability of CDDP, the instructions detailed in the package insert mention that CDDP stability is affected by the concentration of chloride ions in the solution and exposure to light [6]. The instructions also state “mix with saline solution” and “avoid exposing to light if the administration lasts for an extended time period” [6]. Moreover, the supplemental information provided by manufacturers on the Japanese drug interview form of CDDP is limited to the stability testing of one dilution condition, namely, taking 10 mg of the drug to make up 500 mL of solution, monitored under 500 Lux fluorescent light for up to 24 h [7]. Although Zieske et al. have reported that both an increase in pH and exposure to short-wavelength light (350–490 nm) influence the stability of CDDP [8], the details of the conditions that maintain the stability of CDDP have not been clarified.

Therefore, the set dilution conditions of CDDP and the methods employed for light shielding upon CDDP administration vary with different medical institutions. Although there are several regimens comprising CDDP, especially the ESHAP regimen, some have a long treatment time and may need to be shielded appropriately [9]. Therefore, the appropriate shading conditions of CDDP may affect the patient’s therapeutic efficacy and quality of life.

In this study, we analyzed the effects of four shading covers, which are commonly used in clinical settings, on the stability of CDDP. We aimed to determine an effective method for maintaining CDDP stability using tools implementable in clinical settings.

## Methods

### CDDP solution

Randa®, which is a brand-name drug of CDDP (Nippon Kayaku, Tokyo, Japan), was used in this study; it was diluted to 250, 100, and 50 μg/mL using saline (Otsuka Pharmaceutical Factory, Tokushima, Japan). The container (Otsuka Pharmaceutical Factory) used in this study was made of polyethylene. Previous studies have reported that CDDP is not affected by degradation using a polyethylene container [10–13].

### Illuminance conditions

A 1000-Lux fluorescent lamp was used the lighting source. The reason for using this level of illuminance was to ensure exposure to more severe conditions than those used in the Randa® stability test (500 Lux) [7], after considering the general recommendations of lighting levels under the Japanese Industrial Standard (JIS Z9110–2010) for insurance-system-affiliated medical institutions (patient rooms: 100 Lux, hallways: 200 Lux) [14].

### Shading conditions

Four shielding conditions were tested: aluminum foil (Al), brown shading cover (BSC; Terumo Corporation, Tokyo, Japan), yellow shading cover (YSC; Safemick®, JMS Co. Ltd., Hiroshima, Japan), and milky-white anti-exposure cover (MAC; Chemo Cover®, Palmedical Co., Tokyo, Japan), along with no shading cover (NSC) as control.

### Measurement of light transmittance/quantification and pH testing of CDDP

To study the relationship between the wavelength and light transmittance of the four light-shading covers for CDDP stability, we used a UV-visible spectroscope, UV-1850 (Shimadzu, Kyoto, Japan). The transmittance of the light-shading covering a wide range of wavelength (200–800 nm) was monitored. For quantification and pH testing of CDDP in a timely manner, we collected the CDDP samples at 0, 3, 6, 24, 48, 72, 96, and 120 h. Quantification and pH testing of CDDP was performed according to the Japanese pharmacopoeia seventeenth edition (JP17). (Additional file 1: Fig. S1 and Additional file 2: Fig. S2).

## Results

**Fig. 1:**
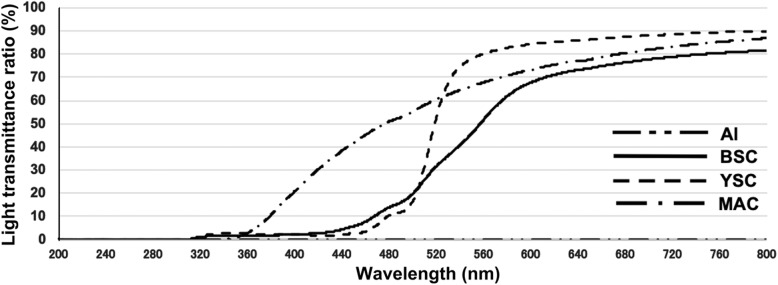
Light transmittance properties of the shielding cover

Figure 1 shows the transmission characteristics of each shading cover, namely, Al, BSC, YSC, and MAC, in the wavelength range of 200–800 nm. A previous study has reported that light absorption in the ca. 250–450 nm region results in cisplatin decomposition [15]. Although we measured a wide range of wavelengths to confirm the characteristics of each shading cover, we particularly focused on the 300–450 nm region on the basis of this previous study. Al could not completely transmit light at all wavelengths. BSC started to show some light transmittance at 440 nm. Similarly, YSC started to show some light transmittance at 440 nm. The MAC started to show some light transmittance with a slow increase at 340 nm; the transmittance reached approximately 40% at 440 nm. Therefore, we found that the shading covers exhibited various different characteristics regarding their transmittance.

To investigate the usefulness of each shading cover, using HPLC we measured the amount of remaining CDDP over 120 h for three concentrations: 250 μg/mL (high), 100 μg/mL (intermediate), and 50 μg/mL (low). First, under the non-shading (NSC) conditions with a high concentration, the amount of remaining CDDP decreased in accordance with a first-order reaction, because photodegradation of CDDP followed first-order kinetics as indicated by Additional file 3: Fig. S3 and Additional file 4: Fig. S4. Under the MAC conditions with a high concentration, the remaining CDDP also decreased in a time-dependent manner, indicating that the MAC could not suppress the degradation of CDDP (Additional file 4: Fig. S4). On the other hand, there was no change in the amount of remaining CDDP under the shading conditions using Al, BSC and YSC with a high concentration. These results indicated that Al, BSC, and YSC could completely suppress the degradation of CDDP (Table 1). The trends for intermediate and low concentration levels were similar to those for high concentration levels regarding the amount of remaining CDDP and degradation rate constant for each shading cover (Table 1 and Additional file 5: Table S1).

**Table 1:** The remaining CDDP and pH increase in a time-dependent manner under each shading condition

Shading condition	CDDP Conc.	Storage time (h)							
		0	3	6	24	48	72	96	120
Al	250 μg/mL	100 ± 0.0 (3.91 ± 0.05)	101.7 ± 1.1 (3.92 ± 0.04)	102.5 ± 1.2 (3.89 ± 0.02)	101.5 ± 1.4 (3.76 ± 0.11)	101.9 ± 1.7 (3.92 ± 0.08)	101.1 ± 1.2 (3.90 ± 0.11)	101.5 ± 2.0 (3.91 ± 0.07)	101.0 ± 1.0 (3.85 ± 0.07)
	100 μg/mL	100 ± 0.0 (4.30 ± 0.06)	101 ± 1.2 (4.31 ± 0.04)	101.8 ± 1.8 (4.30 ± 0.01)	100.3 ± 2.7 (4.19 ± 0.09)	101.8 ± 1.6 (4.32 ± 0.06)	100.8 ± 2.8 (4.31 ± 0.09)	101.6 ± 1.5 (4.26 ± 0.12)	100.6 ± 2.9 (4.27 ± 0.03)
	50 μg/mL	100 ± 0.0 (4.60 ± 0.03)	102.2 ± 0.7 (4.61 ± 0.03)	100.8 ± 0.3 (4.61 ± 0.02)	102.0 ± 1.4 (4.52 ± 0.12)	100.0 ± 0.4 (4.62 ± 0.08)	101.2 ± 2.3 (4.59 ± 0.10)	101.5 ± 1.2 (4.63 ± 0.05)	100.9 ± 2.3 (4.61 ± 0.01)
BSC	250 μg/mL	100 ± 0.0 (3.91 ± 0.06)	100.6 ± 0.5 (3.93 ± 0.04)	100.3 ± 0.2 (3.90 ± 0.02)	99.4 ± 2.0 (3.79 ± 0.17)	100.2 ± 0.2 (3.91 ± 0.11)	100 ± 0.6 (3.91 ± 0.10)	99.8 ± 0.5 (3.92 ± 0.06)	99.3 ± 1.4 (3.88 ± 0.07)
	100 μg/mL	100 ± 0.0 (4.30 ± 0.05)	101.2 ± 0.4 (4.29 ± 0.05)	102.2 ± 0.7 (4.30 ± 0.01)	101.1 ± 0.1 (4.18 ± 0.10)	100.6 ± 1.2 (4.32 ± 0.08)	101.8 ± 0.8 (4.29 ± 0.09)	100.8 ± 0.8 (4.30 ± 0.09)	100.8 ± 1.9 (4.32 ± 0.03)
	50 μg/mL	100 ± 0.0 (4.61 ± 0.04)	99.7 ± 1.6 (4.60 ± 0.04)	100.3 ± 1.2 (4.62 ± 0.02)	100.4 ± 1.8 (4.51 ± 0.13)	100.4 ± 2.6 (4.64 ± 0.07)	101.1 ± 2.9 (4.59 ± 0.11)	100.8 ± 1.3 (4.63 ± 0.08)	97.5 ± 1.4 (4.65 ± 0.01)
YSC	250 μg/mL	100 ± 0.0 (3.91 ± 0.05)	102.2 ± 1.0 (3.92 ± 0.04)	101.0 ± 1.0 (3.91 ± 0.01)	100.2 ± 1.9 (3.79 ± 0.16)	100.0 ± 2.2 (3.95 ± 0.06)	100.7 ± 1.8 (3.95 ± 0.09)	100.2 ± 2.2 (3.92 ± 0.08)	100.2 ± 2.9 (3.94 ± 0.06)
	100 μg/mL	100 ± 0.0 (4.31 ± 0.04)	101.4 ± 0.7 (4.31 ± 0.05)	101.5 ± 0.8 (4.30 ± 0.02)	99.7 ± 1.1 (4.20 ± 0.13)	101.0 ± 0.4 (4.31 ± 0.09)	100.1 ± 0.9 (4.33 ± 0.10)	99.9 ± 0.1 (4.38 ± 0.10)	100.9 ± 1.0 (4.36 ± 0.04)
	50 μg/mL	100 ± 0.0 (4.62 ± 0.03)	99.9 ± 1.1 (4.60 ± 0.03)	99.0 ± 1.4 (4.62 ± 0.02)	98.6 ± 2.1 (4.51 ± 0.14)	97.8 ± 1.1 (4.64 ± 0.06)	98.8 ± 3.1 (4.64 ± 0.09)	98.1 ± 2.0 (4.63 ± 0.10)	97.6 ± 1.0 (4.66 ± 0.02)
MAC	250 μg/mL	100 ± 0.0 (3.91 ± 0.03)	100.2 ± 0.8 (3.96 ± 0.04)	99.3 ± 2.0 (3.95 ± 0.01)	94.3 ± 2.0 (4.06 ± 0.19)	^a^88.9 ± 1.3 (4.77 ± 0.12)	^a^82.9 ± 1.4 (5.97 ± 0.21)	^b^76.0 ± 2.0 (6.50 ± 0.17)	^b^70.8 ± 1.3 (6.85 ± 0.09)
	100 μg/mL	100 ± 0.0 (4.30 ± 0.03)	100.8 ± 1.6 (4.35 ± 0.04)	99.0 ± 1.4 (4.35 ± 0.03)	95.0 ± 1.8 (4.52 ± 0.18)	^a^87.9 ± 1.8 (5.23 ± 0.08)	^a^83.6 ± 1.4 (6.04 ± 0.12)	^b^76.8 ± 2.0 (6.50 ± 0.03)	^b^70.0 ± 2.7 (6.77 ± 0.05)
	50 μg/mL	100 ± 0.0 (4.63 ± 0.02)	100.4 ± 1.2 (4.63 ± 0.04)	98.5 ± 0.6 (4.67 ± 0.03)	94.6 ± 2.4 (4.77 ± 0.14)	^a^89.7 ± 1.8 (5.33 ± 0.09)	^a^83.6 ± 1.8 (5.91 ± 0.15)	^b^76.6 ± 4.0 (6.32 ± 0.06)	^b^71.5 ± 2.9 (6.54 ± 0.03)
NSC	250 μg/mL	100 ± 0.0 (3.94 ± 0.05)	100.2 ± 2.0 (3.96 ± 0.06)	98.6 ± 1.6 (3.96 ± 0.02)	94.7 ± 1.2 (4.11 ± 0.21)	^a^85.3 ± 4.5 (4.86 ± 0.23)	^a^82.4 ± 1.4 (6.25 ± 0.21)	^b^75.0 ± 1.0 (6.84 ± 0.07)	^c^68.7 ± 0.4 (7.11 ± 0.11)
	100 μg/mL	100 ± 0.0 (4.31 ± 0.02)	99.9 ± 1.1 (4.36 ± 0.02)	98.3 ± 1.4 (4.36 ± 0.03)	92.6 ± 1.1 (4.53 ± 0.13)	^a^84.0 ± 5.7 (5.31 ± 0.12)	^a^81.3 ± 0.4 (6.21 ± 0.14)	^b^74.9 ± 1.5 (6.60 ± 0.21)	^c^68.9 ± 1.9 (6.83 ± 0.05)
	50 μg/mL	100 ± 0.0 (4.61 ± 0.06)	102.1 ± 2.3 (4.66 ± 0.03)	98.3 ± 1.3 (4.68 ± 0.03)	93.8 ± 1.5 (4.79 ± 0.08)	^a^86.2 ± 3.8 (5.40 ± 0.11)	^a^83.1 ± 2.5 (6.03 ± 0.18)	^b^77.4 ± 0.6 (6.39 ± 0.02)	^c^69.2 ± 2.3 (6.63 ± 0.03)

Data of the remaining CDDP (pH increase) represent the mean with SD of three independent samples. ^a^ < 90%, ^b^ < 80%, and ^c^ < 70% shows the remaining CDDP compared to 0 h data. Al: aluminum foil, *BSC* brown shading cover, *YSC* yellow shading cover, *MAC* milky-white anti-exposure cover, and *NSC* no shading cover

## Discussion

In this study, to explore the appropriate shading conditions of CDDP, we investigated each wavelength and transmittance using different shielding covers, namely, Al, BSC, YSC, MAC, and NSC. Under each shading condition and a 1000-Lux fluorescent lamp, we examined the amount of remaining CDDP and the pH change. Karbownik et al. previously reported that CDDP is stable in saline for 30 days as long as it is not exposed to light [16]. Our results also showed that the amount of remaining CDDP decreased in a time-dependent manner under NSC conditions. Because there was no a difference in transmittance under BSC and YSC conditions at 440 nm, there was no change in the amount of remaining CDDP. Alternatively, since BSC/YSC started to show some light transmittance at 440 nm and the transmittance of MAC reached approximately 40% at 440 nm, there was a difference in the amount of remaining CDDP between BSC/YSC and MAC. Consistent with a previous study [15], these abovementioned results might indicate that light with a wavelength of ≤ ca. 440 nm reduces the amount of remaining CDDP.

The drug interview form of Randa® Inj. indicates that CDDP is stable when the pH is < 6.89 [7]. Therefore, a change in pH can be considered as an indicator of the stability. The package inserts of several pharmaceutical companies manufacturing CDDP in Japan report the use of a pH adjuster and hydrochloric acid as additives [17–19]. These suggest that the solutions should be kept acidic to maintain the stability of CDDP. To support the results regarding the amount of remaining CDDP, we next examined the increase of pH under each shading cover. Consistent with the results showing a decrease in CDDP levels, we observed that the pH in the solution gradually increased in a time-dependent manner under NSC and MAC conditions. Moreover, because the amount of remaining CDDP was maintained under Al, BSC, and YSC conditions, there was no increase in pH. Taken together, these results demonstrated that the relationship between the remaining CDDP and pH change was matched partially but not completely. Therefore, we found that CDDP degradation was only one factor that increased the pH (Additional file 6: Fig. S5).

Considering the opacity of each cover, we suggest that BSC and YSC are useful and effective for minimizing CDDP photodegradation in a clinical setting. In particular, the use of BSC or YSC should be recommended when CDDP administration exceeds 6 h. Alternatively, although MAC has lower shielding capability than the other shading covers (Table 1), considering the need to prevent the exposure of CDDP, BSC + MAC or YSC + MAC might be also recommended.

The obtained results also indicate the alternatives for preparing, storing, and administering CDDP in clinical facilities, making the treatment schedule more flexible.

## Conclusion

Cumulatively, the findings obtained in this study indicate that the use of appropriate shielding covers, such as BSC or YSC, would prevent the decrease in CDDP under fluorescent lighting and might contribute to achieving full therapeutic effect.

## Supplementary information

**Additional file 1 : Figure S1.** HPLC calibration curve of CDDP.

**Additional file 2 : Figure S2.** Chromatogram used for the quantification of CDDP.

**Additional file 3 : Figure S3.** The amount of remaining CDDP in a time-dependent manner of NSC 250 (a), 100 (b) and 50 (c) μg/mL. Data represent the first-order reaction and degradation rate constant.

**Additional file 4 : Figure S4.** The amount of remaining CDDP in a time-dependent manner of MAC 250 (a), 100 (b) and 50 (c) μg/mL. Data represent the first-order reaction and degradation rate constant.

**Additional file 5 : Table S1.** Degradation rate constant of the first-order reaction under each CDDP concentration under MAC and NSC.

**Additional file 6 : Figure S5.** The amount of remaining CDDP (●) and pH (▲) in a time-dependent manner of NSC 250 μg/mL. These values represent the mean with SD of three independent samples.

## Data Availability

All the data generated or analyzed in this study are included in the published article.

## Abbreviations

BSC: Brown shading cover
CDDP: Cisplatin
HPLC: High-performance liquid chromatography
MAC: Milky-white anti-exposure cover
NSC: No shading cover
RSD: Relative standard deviation
TPN: Theoretical plate number
YSC: Yellow shading cover

## References

[1] Dasari S, Tchounwou PB (2014). Cisplatin in cancer therapy: molecular mechanisms of action. Eur J Pharmacol.

[2] Group IALCTC (2004). Cisplatin-based adjuvant chemotherapy in patients with completely resected non–small-cell lung cancer. N Engl J Med.

[3] Armstrong DK, Bundy B, Wenzel L, Huang HQ, Baergen R, Lele S (2006). Intraperitoneal cisplatin and paclitaxel in ovarian cancer. N Engl J Med.

[4] Wagner AD, Grothe W, Haerting J, Kleber G, Grothey A, Fleig WE (2006). Chemotherapy in advanced gastric cancer: a systematic review and meta-analysis based on aggregate data. J Clin Oncol.

[5] Koriech OM, Shükla V (1981). Dacarbazine (DTIC) in malignant melanoma: reduced toxicity with protection from light. Clin Radiol.

[6] Prescribing information of Randa, 32th edition, Nippon Kayaku Co. Ltd. 2018.

[7] Drug interview form of Randa, 18th edition, Nippon Kayaku Co. Ltd. 2018.

[8] Zieske PA, Koberda M, Hines JL, Knight CC, Sriram R, Raghavan NV (1991). Characterization of cisplatin degradation as affected by pH and light. Am J Hosp Pharm.

[9] Abdel-Malek R, Abbas N, Shohdy KS, Ismail M, Fawzy R, Salem DS (2017). Addition of 3-day aprepitant to ondansetron and dexamethasone for prophylaxis of chemotherapy-induced nausea and vomiting among patients with diffuse large B cell lymphoma receiving 5-day cisplatin-based chemotherapy. J Egypt Natl Canc Inst.

[10] Pujol Cubells M, Prat Aixela J, Girona Brumos V, Duran Pou S, Villaronga FM (1993). Stability of cisplatin in sodium chloride 0.9% intravenous solution related to the container's material. Pharm World Sci.

[11] Rochard E, Barthes D, Courtois P (1992). Stability of cisplatin in ethylene vinylacetate portable infusion-pump reservoirs. J Clin Pharm Ther.

[12] Sewell G (2010). Physical and chemical stability of cisplatin infusions in PVC containers. Eur J Oncol Pharm.

[13] Benaji B, Dine T, Luyckx M, Brunet C, Goudaliez F, Mallevais M (1994). Stability and compatibility of cisplatin and carboplatin with PVC infusion bags. J Clin Pharm Ther.

[14] Japanese Industrial Standard JIS Z 9110:2010 General rules of recommended lighting levels, Japanese Standards Association. 2010.

[15] Macka M, Borak J, Semenkova L, Kiss F (1994). Decomposition of cisplatin in aqueous solutions containing chlorides by ultrasonic energy and light. J Pharm Sci.

[16] Karbownik A, Szalek E, Urjasz H, Gleboka A, Mierzwa E, Grzeskowiak E (2012). The physical and chemical stability of cisplatin (Teva) in concentrate and diluted in sodium chloride 0.9%. Contemp Oncol (Pozn).

[17] Prescribing information of cisplatin, 7th edition, Nichi-Iko Pharmaceutical Co. Ltd. 2018.

[18] Prescribing information of cisplatin, 19th edition, Maruko Pharmaceutical Co. Ltd. 2018.

[19] Prescribing information of cisplatin, 20th edition, Pfizer Inc. 2015.

