# Supplementary figures and images for: Effect of different shielding conditions on the stability of Cisplatin
Source: J Pharm Health Care Sci. 2020 Mar 11;6:3. doi: 10.1186/s40780-020-00163-x (PMC7066770; doi:10.1186/s40780-020-00163-x)

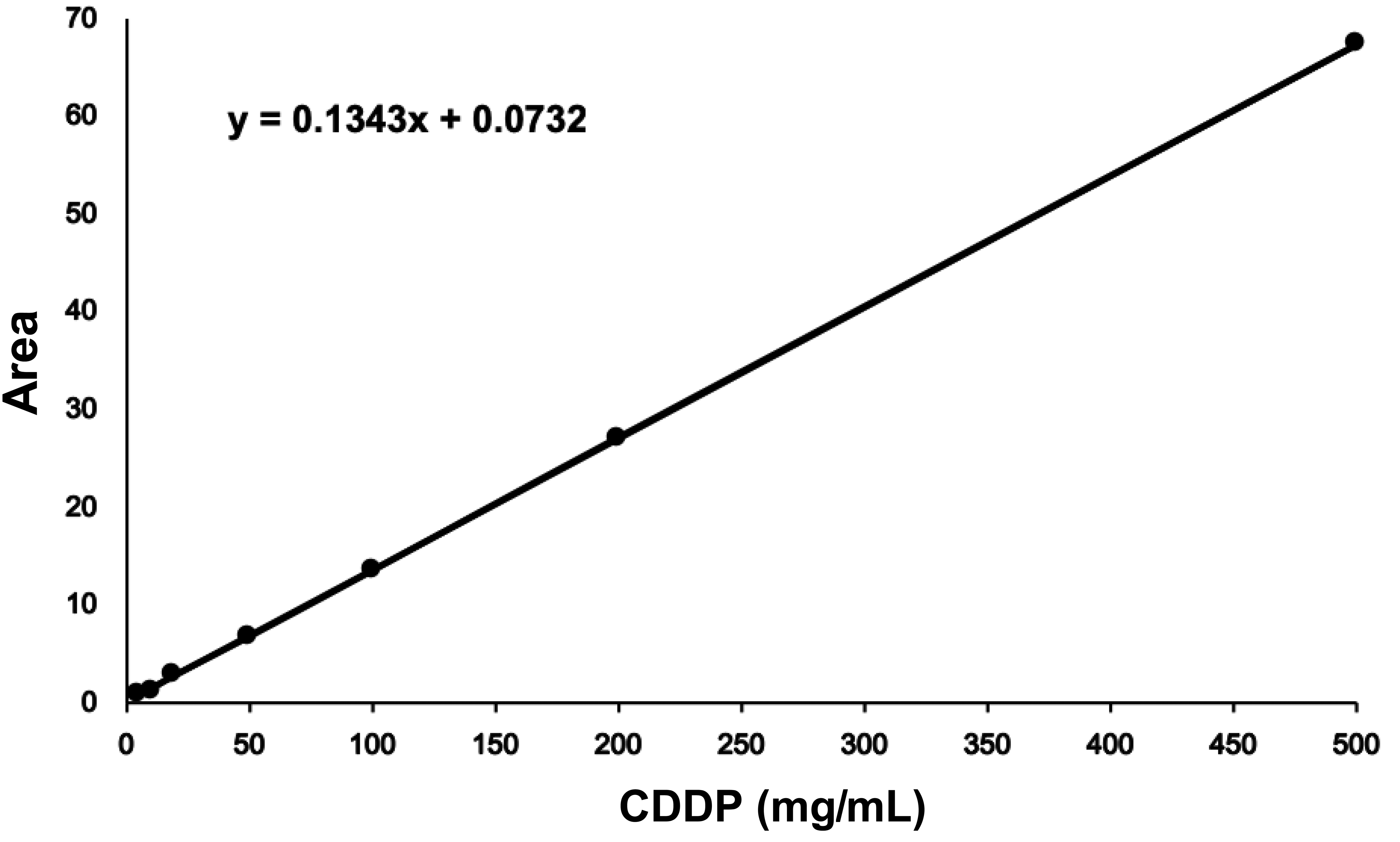

Supplement: Supplementary file 1 — Additional file 1 : Figure S1. HPLC calibration curve of CDDP. [file 40780_2020_163_MOESM1_ESM.tif]

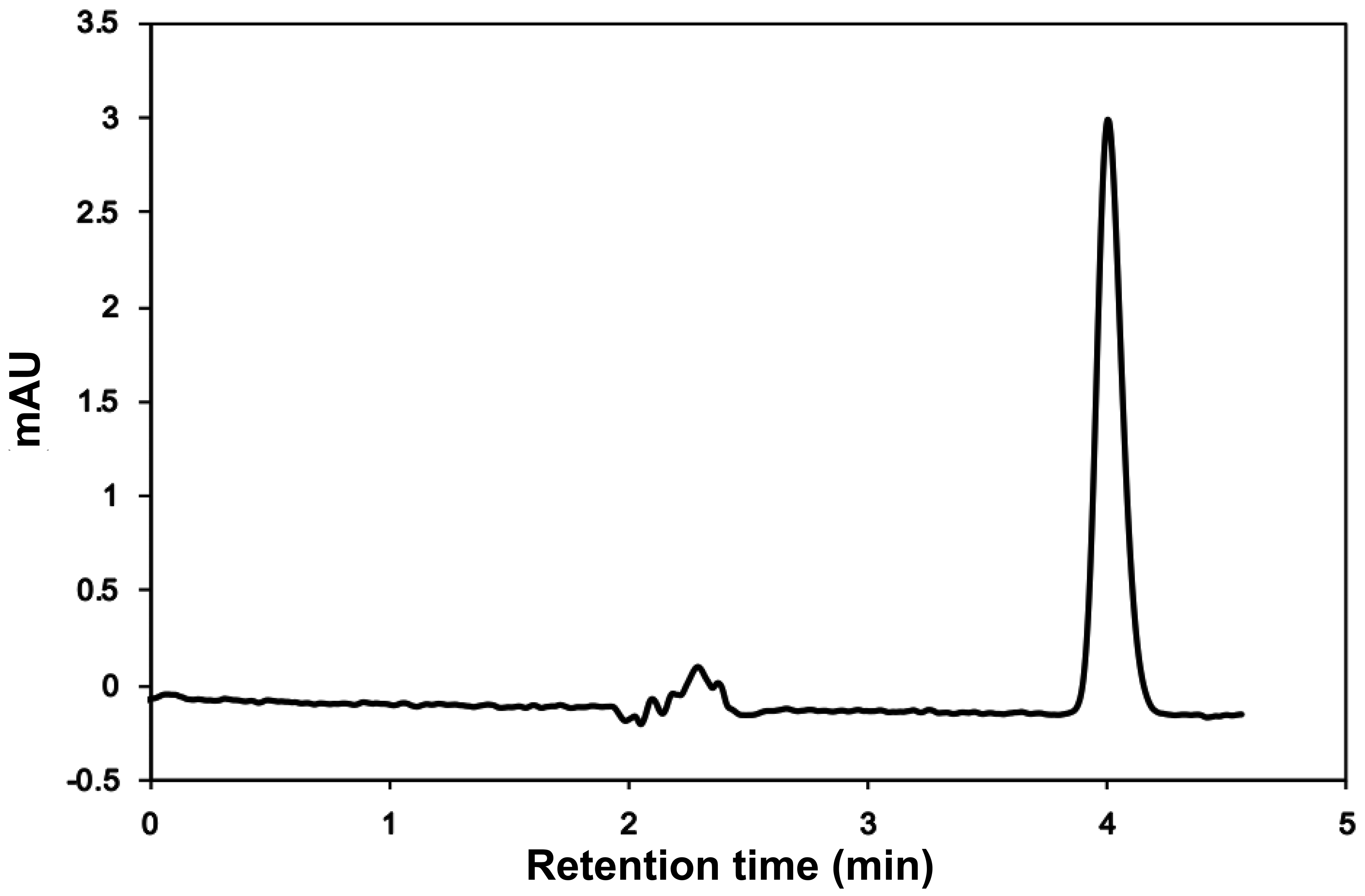

Supplement: Supplementary file 2 — Additional file 2 : Figure S2. Chromatogram used for the quantification of CDDP. [file 40780_2020_163_MOESM2_ESM.tif]

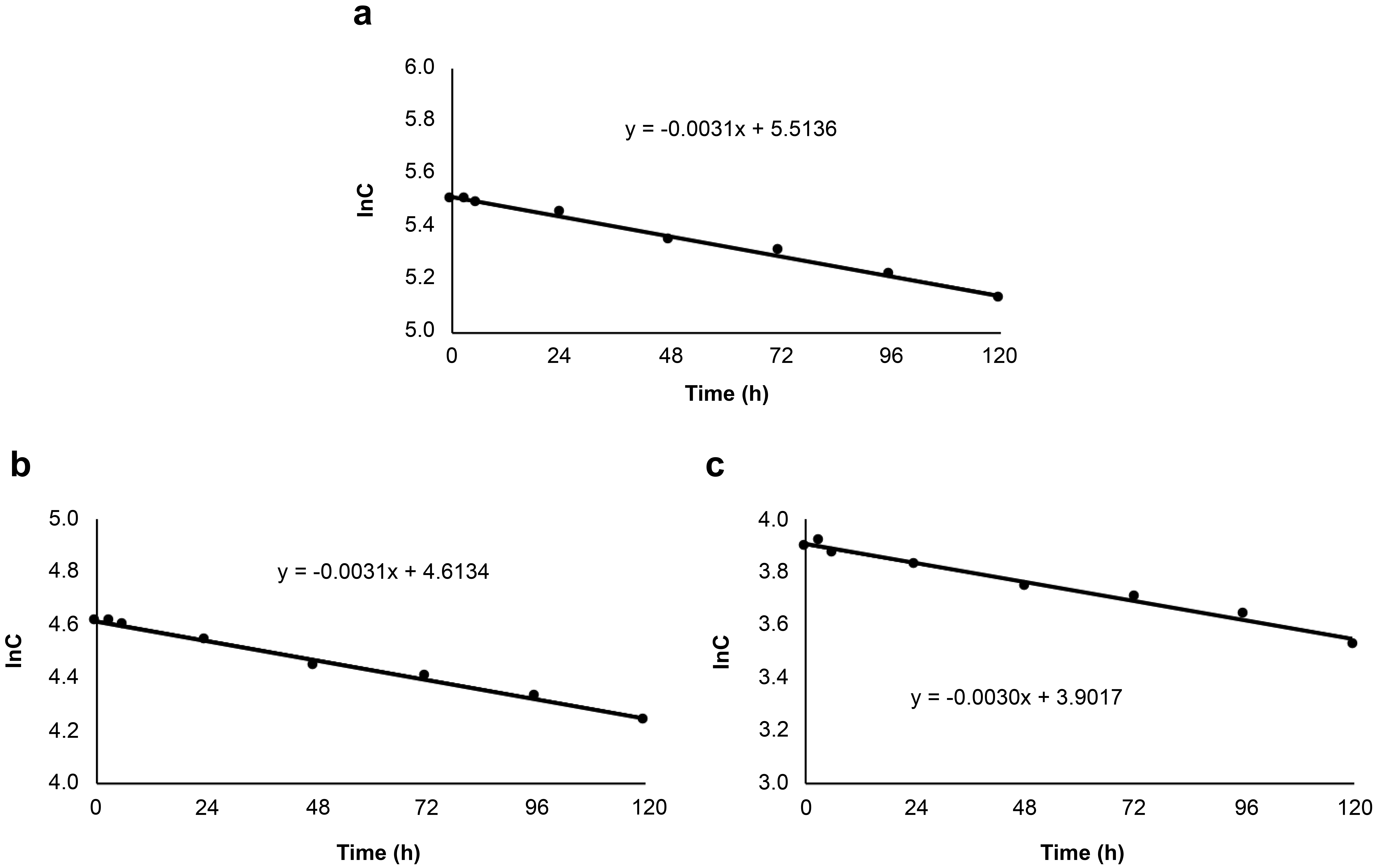

Supplement: Supplementary file 3 — Additional file 3 : Figure S3. The amount of remaining CDDP in a time-dependent manner of NSC 250 (a), 100 (b) and 50 (c) μg/mL. Data represent the first-order reaction and degradation rate constant. [file 40780_2020_163_MOESM3_ESM.tif]

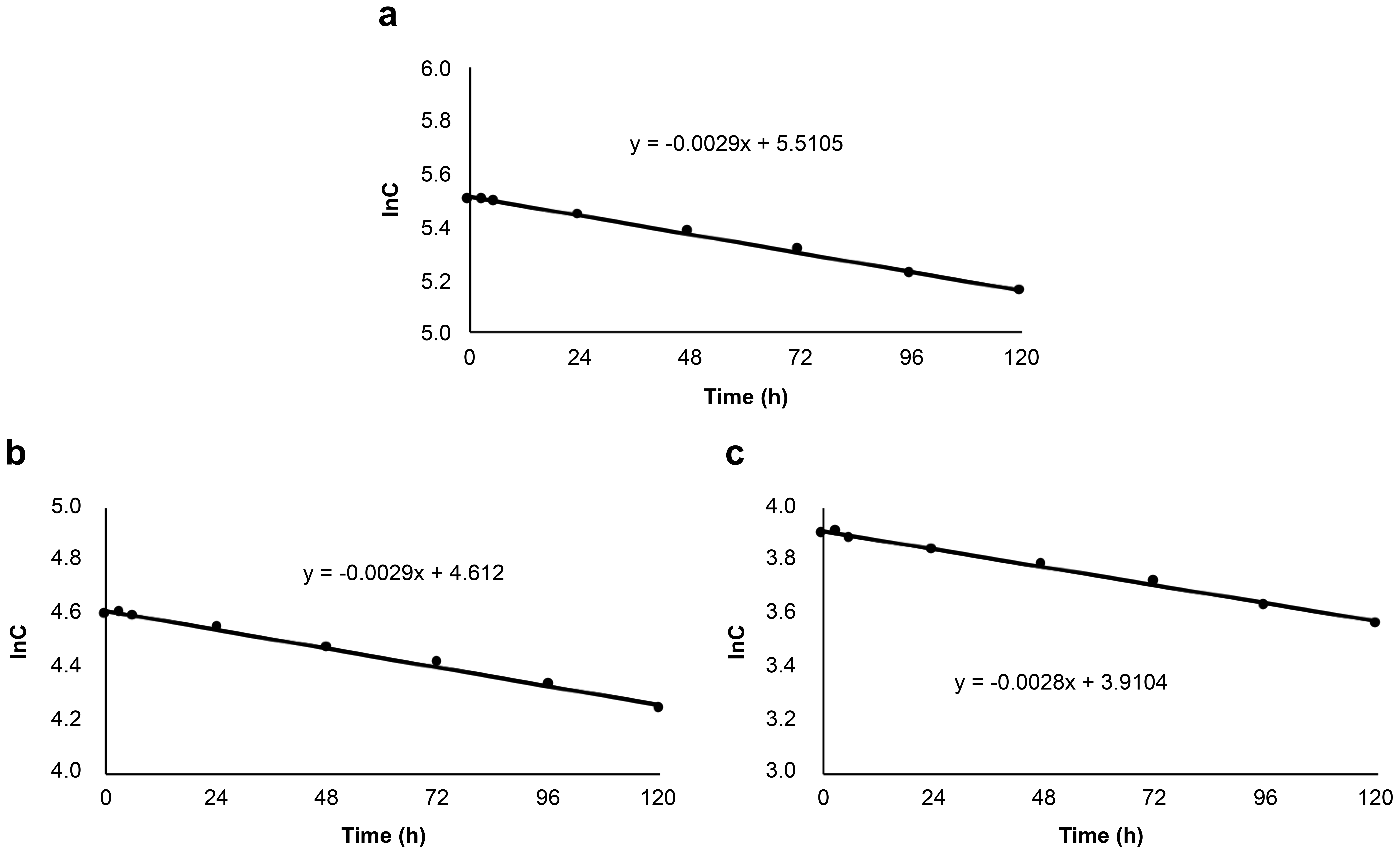

Supplement: Supplementary file 4 — Additional file 4 : Figure S4. The amount of remaining CDDP in a time-dependent manner of MAC 250 (a), 100 (b) and 50 (c) μg/mL. Data represent the first-order reaction and degradation rate constant. [file 40780_2020_163_MOESM4_ESM.tif]

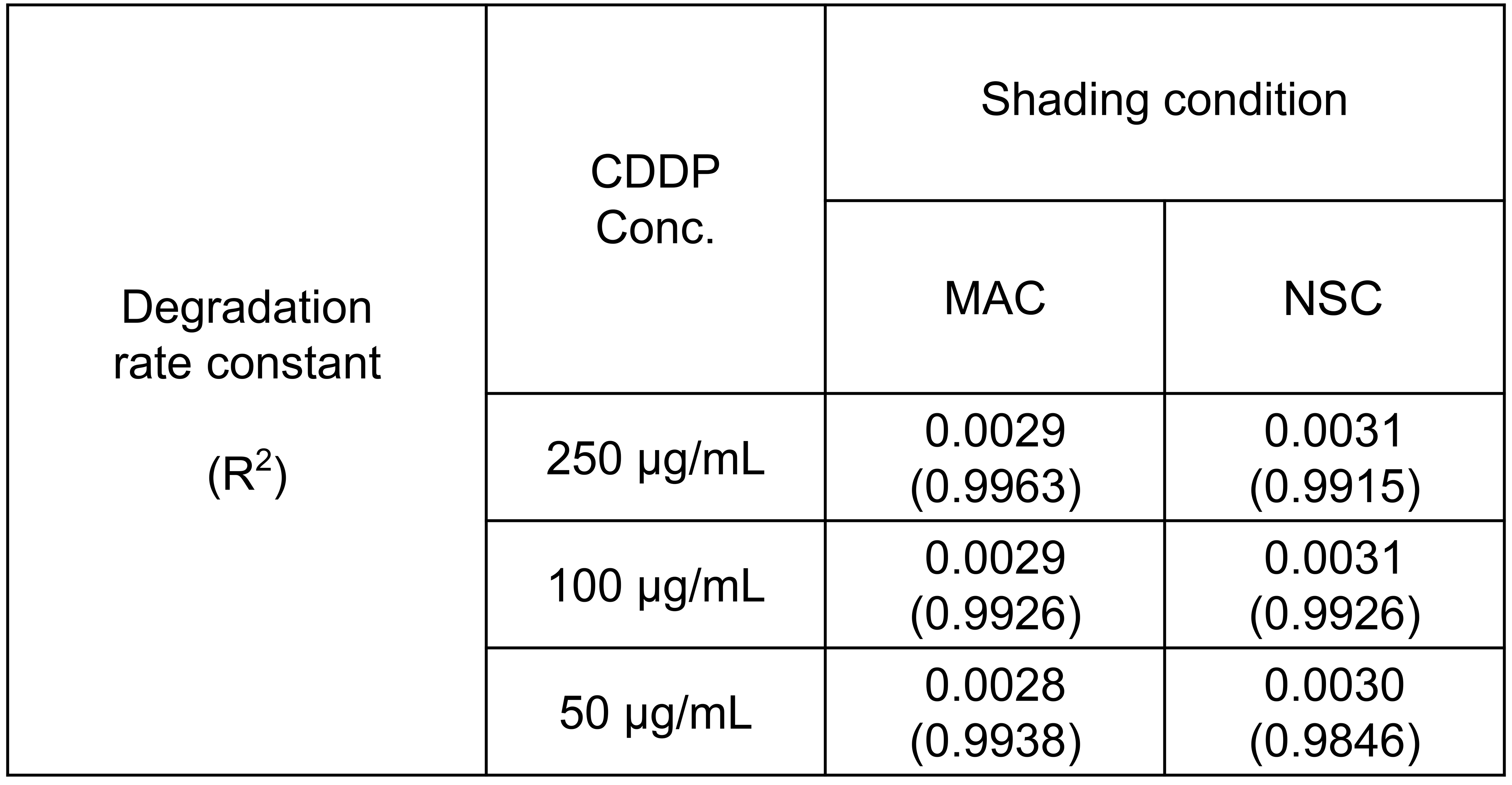

Supplement: Supplementary file 5 — Additional file 5 : Table S1. Degradation rate constant of the first-order reaction under each CDDP concentration under MAC and NSC. [file 40780_2020_163_MOESM5_ESM.tif]

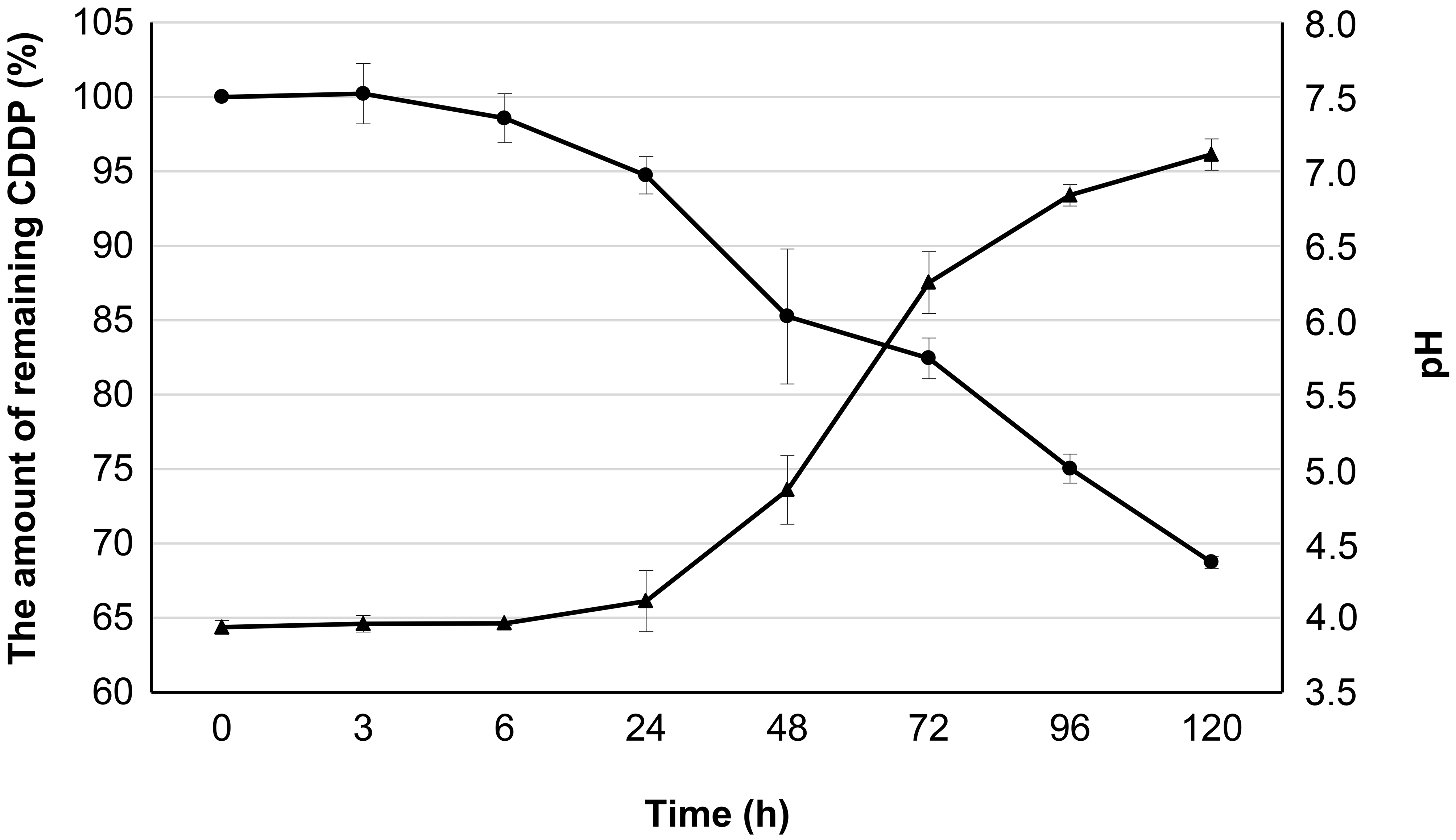

Supplement: Supplementary file 6 — Additional file 6 : Figure S5. The amount of remaining CDDP (●) and pH (▲) in a time-dependent manner of NSC 250 μg/mL. These values represent the mean with SD of three independent samples. [file 40780_2020_163_MOESM6_ESM.tif]
